# Extension of WRF-Chem for birch pollen modelling—a case study for Poland

**DOI:** 10.1007/s00484-020-02045-1

**Published:** 2020-11-11

**Authors:** Małgorzata Werner, Jakub Guzikowski, Maciej Kryza, Małgorzata Malkiewicz, Daria Bilińska, Carsten Ambelas Skjøth, Piotr Rapiejko, Kazimiera Chłopek, Katarzyna Dąbrowska-Zapart, Agnieszka Lipiec, Dariusz Jurkiewicz, Ewa Kalinowska, Barbara Majkowska-Wojciechowska, Dorota Myszkowska, Krystyna Piotrowska-Weryszko, Małgorzata Puc, Anna Rapiejko, Grzegorz Siergiejko, Elżbieta Weryszko-Chmielewska, Andrzej Wieczorkiewicz, Monika Ziemianin

**Affiliations:** 1 Department of Climatology and Atmosphere Protection, University of Wroclaw, Ul. Kosiby 8, 51-621 Wroclaw, Poland; 2 Laboratory of Paleobotany, Department of Stratigraphical Geology, Institute of Geological Sciences, University of Wroclaw, Wroclaw, Poland; 3 School of Science and the Environment, University of Worcester, Worcester, UK; 4 Department of Otolaryngology with Division of Cranio-Maxillo-Facial Surgery, Military Institute of Medicine, Warsaw, Poland; 5 Allergen Research Center Ltd., Warsaw, Poland; 6 Faculty of Natural Sciences, Institute of Earth Sciences, University of Silesia in Katowice, Katowice, Poland; 7 Department of Prevention of Environmental Hazards and Allergology, Medical University of Warsaw, Warsaw, Poland; 8 Department of Immunology and Allergy, Medical University of Lodz, Lodz, Poland; 9 Department of Clinical and Environmental Allergology, Jagiellonian University Medical College, Kraków, Poland; 10 Department of Botany and Plant Physiology, University of Life Sciences in Lublin, Lublin, Poland; 11 Institute of Marine & Environmental Sciences, University of Szczecin, Szczecin, Poland; 12 Pediatrics, Gastroenterology and Allergology Department, University Children Hospital, Bialystok, Poland

**Keywords:** Birch pollen, Pollen season, Air concentrations, Chemical transport model

## Abstract

In recent years, allergies due to airborne pollen allergens have shown an increasing trend, along with the severity of allergic symptoms in most industrialized countries, while synergism with other common atmospheric pollutants has also been identified as affecting the overall quality of citizenly life. In this study, we propose the state-of-the-art WRF-Chem model, which is a complex Eulerian meteorological model integrated on-line with atmospheric chemistry. We used a combination of the WRF-Chem extended towards birch pollen, and the emission module based on heating degree days, which has not been tested before. The simulations were run for the moderate season in terms of birch pollen concentrations (year 2015) and high season (year 2016) over Central Europe, which were validated against 11 observational stations located in Poland. The results show that there is a big difference in the model’s performance for the two modelled years. In general, the model overestimates birch pollen concentrations for the moderate season and highly underestimates birch pollen concentrations for the year 2016. The model was able to predict birch pollen concentrations for first allergy symptoms (above 20 pollen m^−3^) as well as for severe symptoms (above 90 pollen m^−3^) with probability of detection at 0.78 and 0.68 and success ratio at 0.75 and 0.57, respectively for the year 2015. However, the model failed to reproduce these parameters for the year 2016. The results indicate the potential role of correcting the total seasonal pollen emission in improving the model’s performance, especially for specific years in terms of pollen productivity. The application of chemical transport models such as WRF-Chem for pollen modelling provides a great opportunity for simultaneous simulations of chemical air pollution and allergic pollen with one goal, which is a step forward for studying and understanding the co-exposure of these particles in the air.

## Introduction

Exposure to respirable allergenic materials (aeroallergens) from bioaerosols can stimulate the production of antibodies in the human body and cause allergic airway diseases (AAD), such as asthma, and allergic rhinitis (Adhikari et al. 2006). AAD is a serious public health concern worldwide with the most prevalent impacts among children and adolescents (Miguel et al. 2006; Taylor et al. 2007). Pollen allergy is a reaction of the human immune system to certain allergens carried by airborne pollen. Allergic rhinitis affects approximately 10 to 30% of the global population according to the WAO White Book on Allergy: Update 2013 (Pawankar et al. 2013). Symptoms of allergic rhinitis were reported by almost a quarter of respondents in the largest Polish epidemiological study to date (ECAP) (Samoliński et al. 2009). In the general population of Europe, the prevalence of *Betula* (birch) pollen sensitization ranges from 8 to 16% (Biedermann et al. 2019). In a Polish epidemiological study, positive skin prick tests with birch pollen allergens were recorded in 14.9% of the representative population (Samoliński et al. 2014).

In recent years, allergies due to airborne pollen have shown an increasing trend, along with the severity of allergic symptoms in most industrialized countries, while synergism with other common atmospheric pollutants has also been identified as affecting the overall quality of citizenly life (Després et al. 2012). The combined exposure to chemical and biological air pollutants (henceforth, “chemical pollutants” will be called “pollutants”) may strengthen allergenic reactions (Baldacci et al. 2015). In addition, air pollutants may directly interact with airborne pollen grains, affecting their morphological features and altering allergic properties (Behrendt and Becker 2001). Various air pollutants can act as adjuvants to allergenic pollen, thereby increasing the frequency and/or severity of allergic airway diseases (Behrendt and Becker 2001), such that being able to predict the timing of the pollen season relative to peak pollution times is important. The need to consider both pollen and pollutant contents for the epidemiologic evaluation of environmental determinants in respiratory allergies has recently been reported (Schiavoni et al. 2017).

The allergic symptoms usually start with the pollen season and vary in severity. The triggering of allergic reactions is highly correlated with airborne pollen concentration levels. Different individuals may experience symptoms of varying severity for the same level of concentration (Voukantsis et al. 2010). This highlights the need to produce pollen concentration forecasts and disseminate this information in a timely manner to those potentially impacted people, in order to better manage the severity of their allergic reactions.

More than 10 years ago, pollen dispersion was either modelled by the Lagrangian trajectory models such as CALPUFF (Pfender et al. 2006), HYSPLIT (Stach et al. 2007; Hernández-Ceballos et al. 2011) or Finnish Emergency Dispersion Modelling System (SILAM) (Sofiev et al. 2006), or by Gaussian advection-diffusion models such as ADMS (Hunt et al. 2001), Acquilon (Dupont et al. 2006) or METRAS (Schueler and Schlünzen 2006). In the last decade, there has been a growing effort to simulate regional pollen dispersal with more complex Eulerian regional air-quality models. These models include a detailed description of physical and chemical processes in the atmosphere as well as the ability to estimate the co-exposure of air pollution and bioaerosols with the same modelling framework. They also include transport of air pollution and bioaerosols from other countries, while some of them can quantify feedbacks between bioaerosols, air pollution, and weather. However, the application of these models is still complicated because of the high computing costs (even as the problem is getting smaller with increasing capabilities of computers) and their complexity concerning, among others, configuration and the amount of input information. The number of applications for the Eulerian chemical transport model for birch pollen has recently increased, e.g. within the Monitoring of Atmospheric Composition and Climate (MACC, http://www.gmes-atmosphere.eu) project. For the MACC project, seven models, i.e. CHIMERE, EMEP model of EMEP/MSC-W, EURAD-IM, LOTOS-EUROS, MATCH, MOCAGE, and SILAM, with the emission module proposed by Sofiev et al. (2013) were used to simulate birch pollen concentration over Europe (Sofiev et al. 2015). The results were validated for several countries in western and north-eastern Europe but not for Poland. The results showed that Eulerian, regional models have great potential for reproducing the main characteristics of birch pollen season and birch pollen concentrations over Europe.

There are two main modelling approaches used within the Eulerian models—offline and online systems (Baklanov 2010; Baklanov et al. 2014). The online integration of meteorological models and chemical transport models (CTMs) provides an opportunity to use 3D meteorological fields in CTM at each time step. It also allows for considering the impact of air pollution on meteorological processes and then on the atmospheric chemical composition, which is especially relevant for the modelling of chemical compounds (e.g. NO_*x*_ or O_3_). The offline approach is more computationally efficient compared to the online approach—one simulation with the meteorological model can provide input data to many simulations with chemical transport models. A list of the advantages of both the online and offline approaches is given by Baklanov (2010). The application of Eulerian models for pollen modelling within the last 10 years included both offline approach, e.g. with SILAM (Sofiev et al. 2015; Galán et al. 2017; Sofiev 2019) and online approach, e.g. with COSMO-ART (Pauling et al. 2012; Zink et al. 2013) and Enviro-HIRLAM (Kurganskiy et al. 2020).

In this study, we propose the state-of-the-art WRF-Chem model, which is a complex Eulerian meteorological model integrated on-line with atmospheric chemistry. The model has had many applications for air quality (chemical pollutants) simulations in many countries, including those in Europe (Baklanov et al. 2014). Here, we use a combination of the WRF-Chem extended towards birch pollen and the emission module based on parameterization proposed by Sofiev et al. (2013), both of which have not been previously used together. The main question is whether the modelling system based on the WRF-Chem model is able to reproduce birch pollen concentrations for various cases, in terms of pollen productivity and pollen seasons, and provide allergic people reliable information on birch pollen levels. We focus on the threshold concentrations of birch pollen, which cause various allergenic symptoms among impacted people. We evaluate the model results in the context of the main parameters describing the season (e.g. start and end of the season, seasonal pollen integral), and temporal variations in birch pollen concentrations through comparison with daily measurements from 11 stations located in Poland.

## Data and methods

### WRF-Chem model

The Weather Research and Forecasting model with Chemistry (WRF-Chem) has been widely used for calculations of air pollution concentrations in a re-analysis and forecasting mode (Baklanov et al. 2014). The forecasting system of meteorology and air pollution based on WRF-Chem was developed at the University of Wroclaw (Werner et al. 2015), and the results are available at their geoportal (https://powietrze.uni.wroc.pl). The model has many options for physical parameterizations as well as gas-phase chemical mechanisms, photolysis processes, and aerosol schemes. WRF-Chem describes the major processes that are important for atmospheric transport of bioaerosols, such as convective transport, turbulent mixing, and dry and wet deposition. A complete description of the model is given by Grell et al. (2005) and Fast et al. (2006). The model can work with several chemical mechanisms and different aerosol modules, and is widely used in atmospheric transport models including the Model for Simulating Aerosol Interactions and Chemistry (MOSAIC), Modal Aerosol Dynamic model for Europe (MADE), and the bulk aerosol module from GOCART, which was used in this study.

The chemical component of WRF-Chem is fully consistent with the meteorological component (Grell and Baklanov 2011). Hence, both meteorological and air quality components use the same physics schemes for the sub-grid scale transport, the same grid on the horizontal and vertical coordinates, and the same atmospheric transport scheme (advection and diffusion) which preserves air and scalar mass (Tsarpalis et al. 2018). The GOCART module includes algorithms for dry deposition and gravitational settling (Legrand et al. 2018; Ukhov et al. 2020). The gravitational settling parameterization is based on calculation of settling velocity, which depends on particle density and size. The wet deposition parameterization is based on the Jung scheme (Tsarpalis et al. 2018) and includes both in-cloud (rainout) and below-cloud (washout) scavenging.

**Fig. 1:**
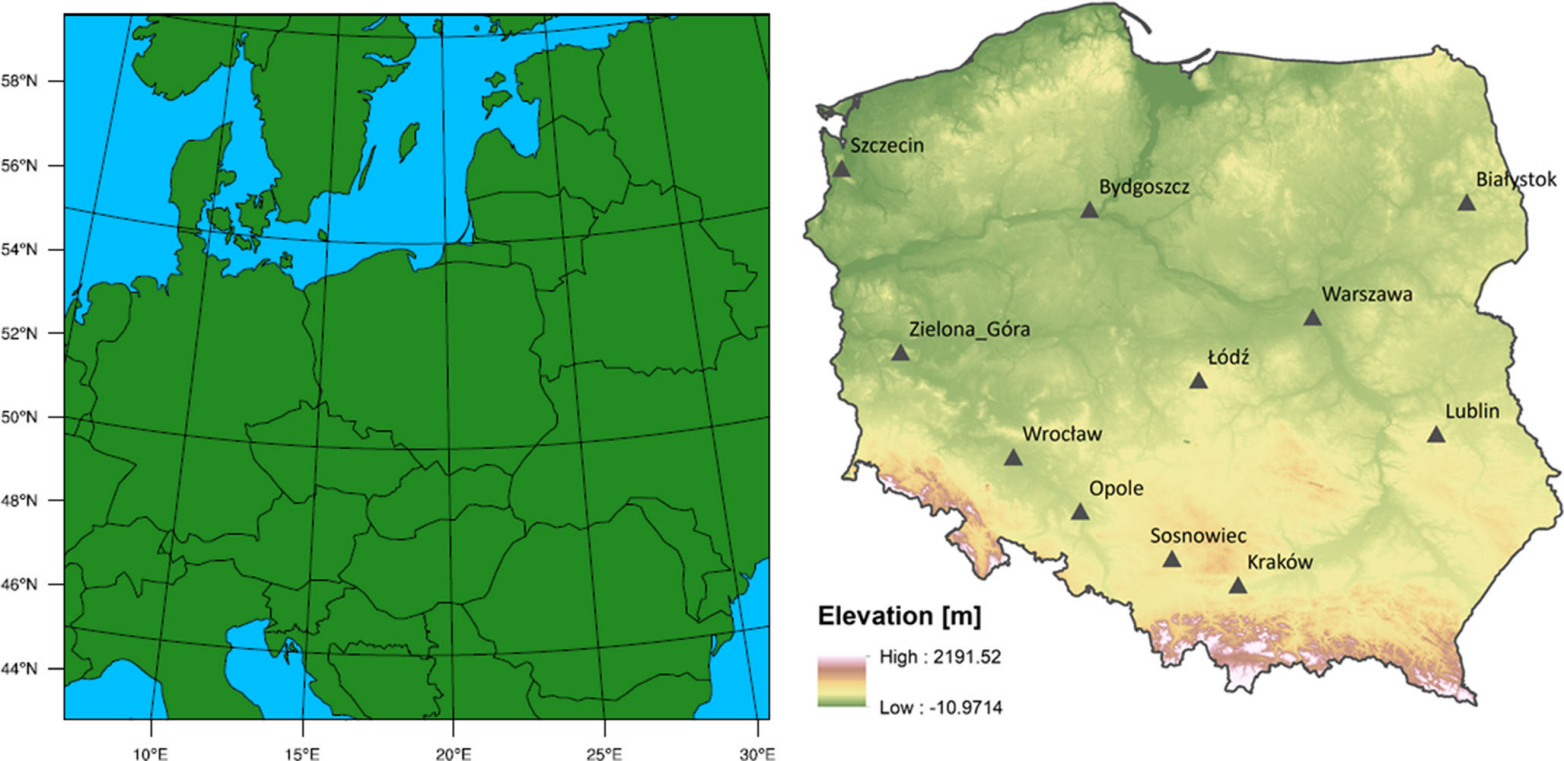
Simulation domain (left) and location of stations in Poland (right)

The computational domain covers Europe at a 12 km × 12 km grid (Fig. 1). Initial and boundary conditions for meteorological fields were obtained from the NCEP FNL Operational Global Analysis data with a horizontal resolution of 1° × 1°, 27 (32 since 11 May 2016) vertical levels, and temporal resolution of 6 h. The data were interpolated to the model grid using the WRF pre-processing system (WPS). Model configurations regarding physical parameters are the same as in Werner et al. (2019a).

### Emission model

**Fig. 2:**
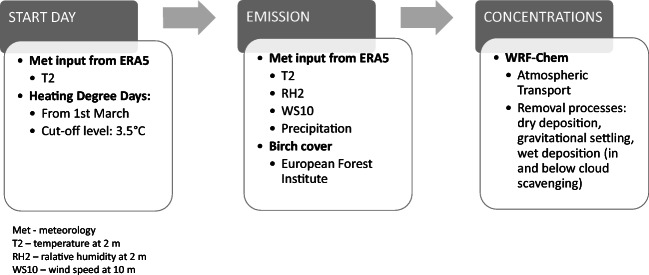
Flow chart of the birch pollen modelling process

Birch pollen emission was calculated based on heating degree day (HDD) threshold parameterization whose original version is described in detail by Sofiev et al. (2013). Below, we provide the main concept of the emission module and input data that were used to calculate emissions for our domain. The flow chart of the modelling process is given in Fig. 2. Meteorological parameters were obtained from the ERA5 reanalysis dataset (https://www.ecmwf.int/). The data covers the Earth with c.a. 30 km × 30 km grid and is available with 1-h temporal resolution. The model assumes that the birch productivity is the same in all years and equal to 10^9^ pollen m^−2^ season^−1^. The emission model calculations were done separately from the model run as a pre-processing step. In the first step, we calculated the sum of daily temperature above a cut-off level (3.5 °C) from the 1st of March. If the calculated sum exceeded the temperature sum thresholds at any grid in the domain, the emission model would then start the calculation of birch pollen emissions for this area. The maps of the temperature sum threshold for the start of the season and cut-off level were taken from the study of Siljamo et al. (2013) and Sofiev et al. (2013). The rate of heat accumulation is the main controlling parameter for pollen emission: the model establishes direct proportionality between the flowering stage and a fraction of heat sum accumulated to date (Linkosalo et al. 2010; Sofiev et al. 2015).

A release flux of pollen grains expressed in the number of grains emitted from 1 m^2^ of birch forest within 1 s was calculated for each model grid cell. Apart from temperature, three meteorology-dependent corrections were applied to the dynamic release rate: wind speed, relative humidity, and precipitation rate. In general, there is no pollen release if the weather is cool, if there is high relative humidity, or if it is rainy. Precipitation and humidity-related corrections are derived from known “prohibiting” thresholds totally suppressing the pollen release (Sofiev et al. 2013). The lower and upper thresholds of relative humidity taken are respectively 50 and 80%; for precipitation, it is 0 and 0.5 mm h^−1^. Strong wind promotes release by up to 50%. In the last step, the calculated emission was multiplied by the fraction of the birch forest for each grid cell provided by the European Forest Inventory (EFI, https://www.efi.int/knowledge/maps/treespecies). The EFI database contains a set of 1 × 1 km^2^ tree species maps showing the distribution of 20 tree species over Europe. The data are based on dendrometric data from 17 countries in Europe. In areas with national forest inventory data, area proportions covered by the 20 species were obtained by compositional kriging. For the rest of Europe, a multinomial logistic regression model was fitted to ICP-level-I plots using various abiotic factors as predictors (soil, biogeographical zones, bioindicators derived from temperature, and precipitation data). Details are given in Brus et al. (2012). The end of the season is described via the open-pocket principle: the flowering continues until the initially available amount of pollen is completely released (Sofiev et al. 2013).

### Study period

The simulations were done for the years 2015 and 2016, which were very different in terms of birch pollen concentrations. The season 2016 in Poland was characterized by high birch pollen concentrations (Weryszko-Chmielewska et al. 2016; Kubik-Komar et al. 2019). The birch annual pollen sums were several times higher compared to 2015. For example, for Wroclaw, it was 3 times higher (Weryszko-Chmielewska et al. 2016), whereas for Lublin, it was up to even 16 times higher (Kubik-Komar et al. 2019). The birch pollen season in 2016 was also influenced by a long-distance transport with air masses from Northern Africa (Puc et al. 2016). Simulations for these 2 years provide an opportunity to verify whether the model is capable of properly simulating birch pollen concentrations at different conditions in terms of level of concentrations. The simulations with the WRF-Chem model were run from the 1st of April to May 15th.

### Observational data and model evaluation

Measured birch pollen concentrations from 11 stations (Fig. 1) were provided by the Allergen Research Centre and cooperating university centres in Poland. The data were gathered using a Burkard or Lanzoni 7-day volumetric pollen trap based on the Hirst design (Hirst 1952) and analyzed following recommendations from the International Association for Aerobiology (Galán et al. 2014). The observations are available as daily mean concentrations expressed as the number of pollen grains per 1 m^−3^ of air (pollen m^−3^).

The modelled hourly birch pollen concentrations were aggregated into daily mean values for comparison with observations. To validate the modelling results, first, we compared modelled and measured values of the primary parameters describing the season, i.e. its start and end and seasonal pollen integral (SPIn). The start and end of the season were calculated as the dates when 5 and 95% of the cumulative seasonal pollen sums of the observed pollen concentrations were respectively reached. The SPIn was calculated as the sum of daily pollen concentrations over the whole birch pollen season (Galán et al. 2017). Afterwards, we then calculated the standard statistical metrics such as the following: mean bias (MB), mean absolute error (MAE), correlation coefficient (R), fractional bias (FB), and fractional absolute error (FAE) (Yu et al. 2006). The equations are provided in Table 1 .

**Table 1:** Definitions of error statistics used in the study. O is for observed values and M is for modelled values

Statistic	Formula	Range of values	Expected value
Mean bias (MB)	$\mathrm{MB}=\frac{1}{N}{\Sigma}_1^N\left({M}_i-{O}_i\right)$	(−Ō, +∞)	0
Mean absolute error (MAE)	$\mathrm{MAE}=\frac{1}{N}{\Sigma}_1^N\mid {M}_i-{O}_i\mid$	(0, +∞)	0
Correlation coefficient (*R*)	$R=\frac{\sum_{i=1}^N\left({M}_i-\overline{M}\right)\left({O}_i-\overline{O}\right)}{{\left\{{\sum}_{i=1}^N{\left({M}_i-\overline{M}\right)}^2{\sum}_{i=1}^N{\left({O}_i-\overline{O}\right)}^2\right\}}^{\frac{1}{2}}}$	(− 1, 1)	1
Fractional bias (FB)	$\mathrm{FB}=\frac{1}{N}{\Sigma}_1^N\ \frac{\left({M}_i-{O}_i\right)}{\left({M}_i+{O}_i\right)/2}$	(− 2, 2)	0
Fractional absolute error (FAE)	$\mathrm{FAE}=\frac{1}{N}{\Sigma}_1^N\ \frac{\mid {M}_i-{O}_i\mid }{\left(\mathrm{M}+{O}_i\right)/2}$	(0,2)	0

In the next step, we checked if the model can simulate high birch pollen concentrations, relevant for allergic individuals. The threshold values of birch pollen concentrations were proposed by Rapiejko et al. (2004) (Table 2). We used these values to convert birch pollen concentrations into a binary event and summarized by a contingency table (Table 3). The following criteria are used: hits (correct forecast and event), misses (observed but not forecasted event), false alarms (forecast but not observed event), and correct rejections (correct forecast of non-event) (Wilks 2011). Based on the contingency table, we prepared the performance diagram, which summarizes and compares the results for the various birch pollen threshold values. The statistics used by the performance diagram are given in Table 4. The results for an ideal model are located in the upper right corner of the performance diagram.

**Table 2:** The concentration of birch pollen and the corresponding clinical symptoms

Symptoms	Birch pollen (pollen m^−3^)
First symptoms	20
Symptoms in all subjects	75
Severe symptoms	90
Symptoms of dyspnea	155

**Table 3:** Contingency table. Counts a, b, c, and d are the total number of hits, false alarms, misses, and correct rejections, respectively

	Event observed	
Event forecast	Yes	No
Yes	a	b
No	c	d

**Table 4:** Statistics used by the performance diagram

Statistic	Equation
Probability of detection	POD = *a*/(*a* + *c*)
Success ratio	SR = 1 − (*b*/(*a* + *b*))
Bias	BIAS = (*a* + *b*)/(*a* + *c*)
Critical Success Index (threat score)	CSI = *a*/(*a* + *b* + *c*)

## Results

**Fig. 3:**
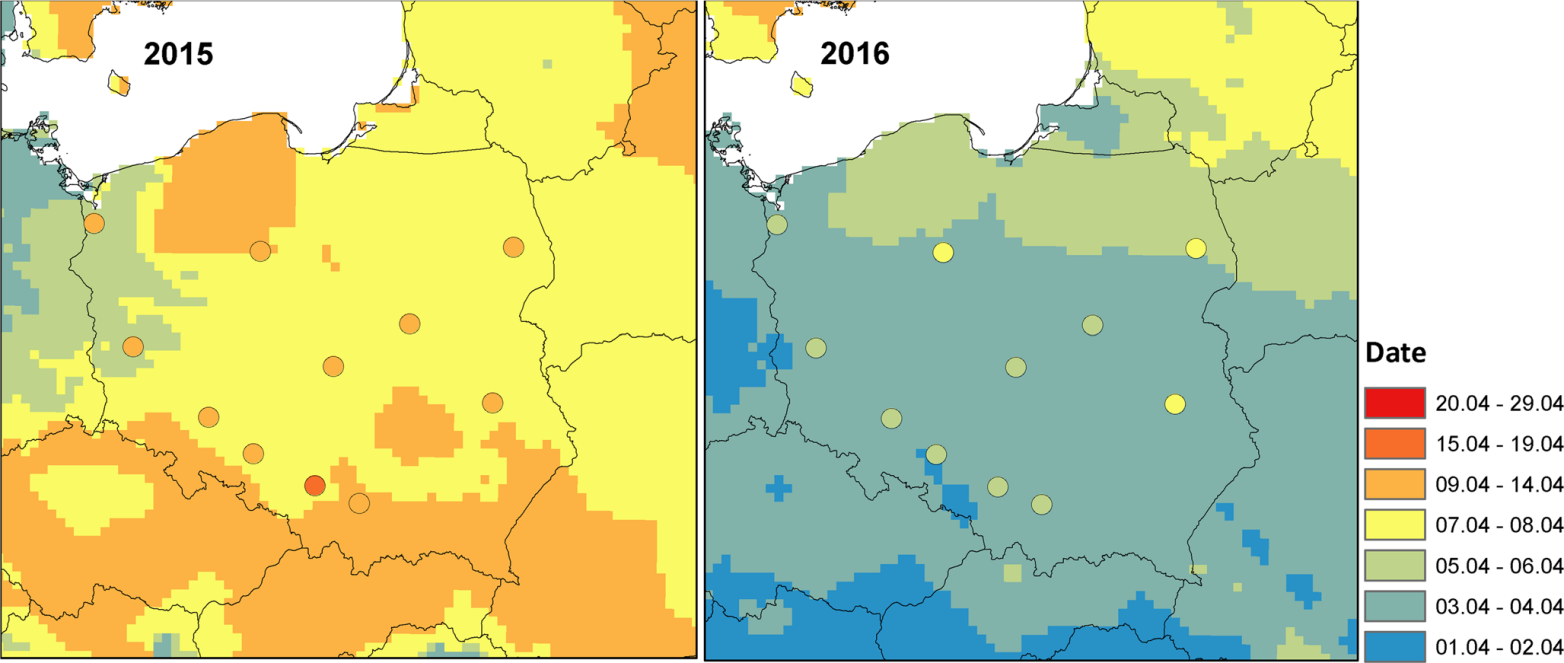
Modelled and observed (signed by dots) date of start (date of 5% of the cumulative seasonal total) of the birch pollen season for the year 2015 (left) and 2016 (right)

The start of the birch pollen season calculated with WRF-Chem varies from April 03 to April 12 and from April 02 to April 07 for the years 2015 and 2016, respectively (Fig. 3). For most of the domain, the season starts between April 07 and 09 for the year 2015 and between April 03 and 04 for the year 2016. In general, the season starts earlier in the west and later in the north and north-east Poland which is in agreement with the general pattern of the vegetation season (Wypych et al. 2017). A later start of the season was calculated for the mountainous area in the south of the country for the year 2015, e.g. Tatra Mountains. This later start in the mountains is not noticed in the year 2016. A comparison of the modelled and observed start of the season shows that at all stations and in both years, the model calculates the start of the season earlier than the one calculated from observations. The mean difference between the model and observations is 3 days in 2015 and 2 days in 2016.

**Fig. 4:**
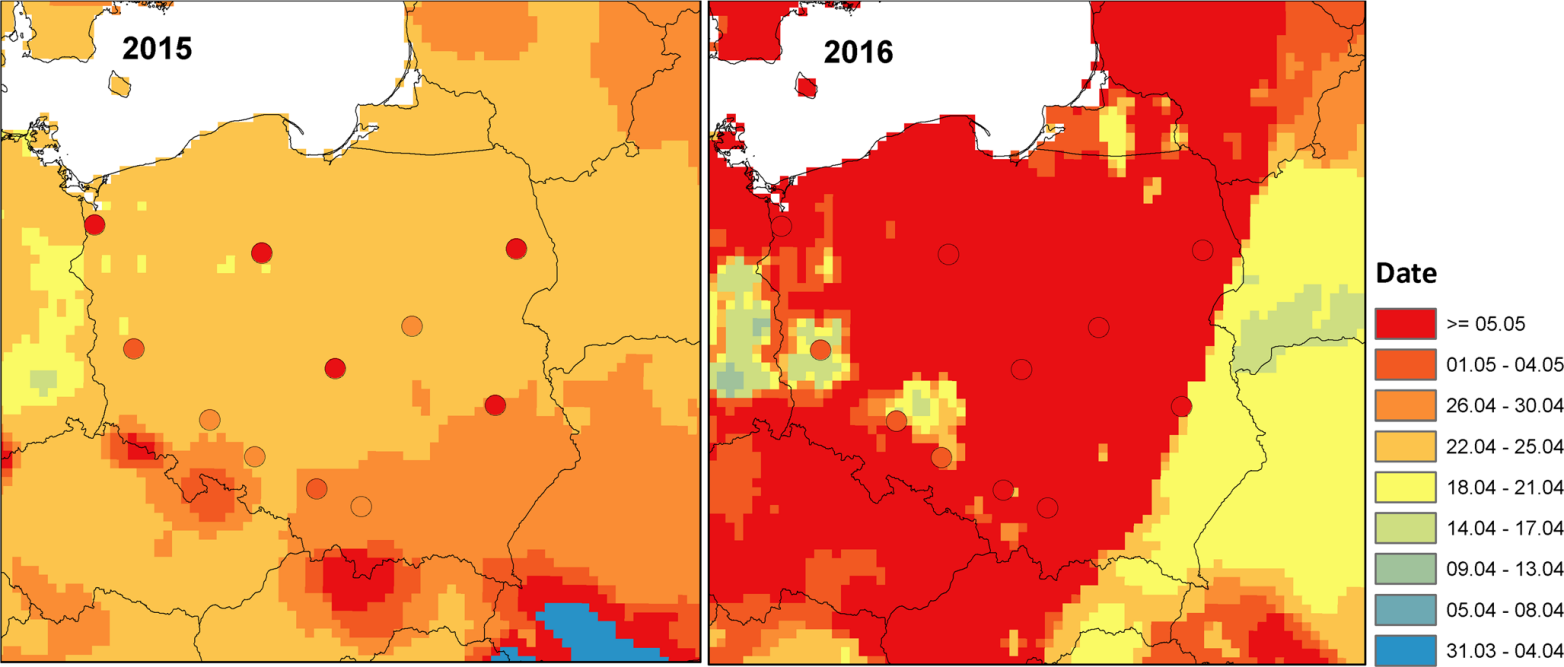
Modelled and observed (signed by dots) date of end (date of 95% of the cumulative seasonal total) of the birch pollen season for the year 2015 (left) and 2016 (right)

The end of the birch pollen season calculated with WRF-Chem is more stretched over time than the start of the season (Fig. 4). It varies from April 21 to May 08 in 2015 and from April 12 to May 06 in 2016. The season ends at the latest in the mountainous regions of southern Poland for the year 2015, whereas in 2016, over a large area (including mountainous area), it ends on May 06. The model predicts the end of the season earlier than that calculated from observations. The mean difference between the model and observations is 9 days in 2015 and 4 days in 2016. The smallest difference between the model and observations (3–4 days) for both years is in southern Poland (Wrocław, Opole, Kraków).

**Fig. 5:**
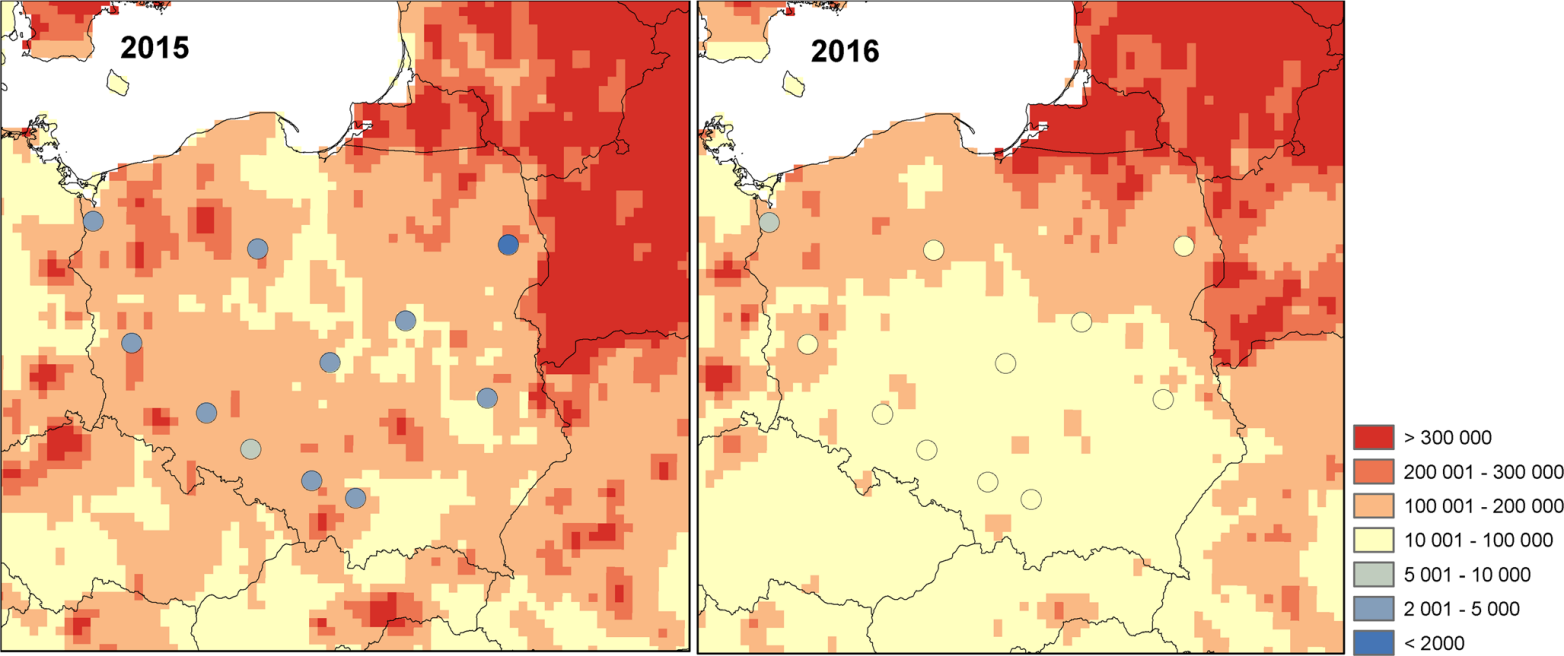
Modelled and observed (signed by dots) seasonal pollen integral over Poland for the year 2015 and 2016

According to the model, the seasonal pollen integral over Poland varies from 80,000 to 450,000 pollen m^−3^ in 2015 and from 26,000 to 350,000 pollen m^−3^ in 2016 (Fig. 5). The highest values cover especially north-east, south-east, and western Poland, which correspond to the high contribution of birch trees in land use. The observed SPIn is overestimated by the model for the year 2015 and underestimated for the year 2016. The mean difference between the model and observations is 24,000 and − 18,000 g m^−3^ for the year 2015 and 2016, respectively.

**Fig. 6:**
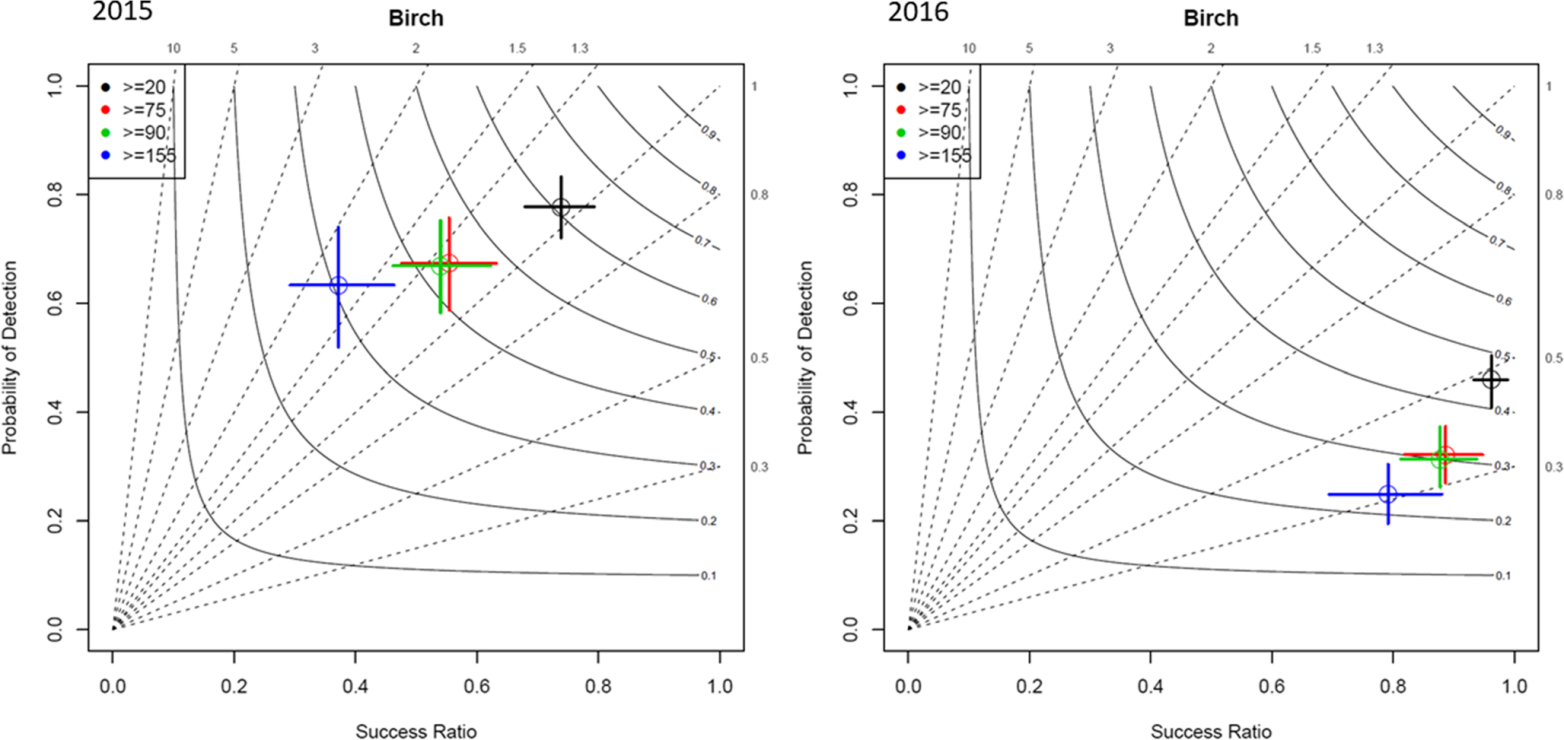
Performance diagram for the threshold birch pollen concentrations based on Polish stations for the season 2015 (left) and 2016 (right). Threshold concentrations: (1) 20 pollen m^−3^—first symptoms, (2) 75 pollen m^−3^—symptoms in all subjects, (3) 90 pollen m^−3^—severe symptoms, and (4) 155 pollen m^−3^—symptoms of dyspnea

The performance diagrams are presented in Fig. 6. The figures show that for 2015, the WRF-Chem model is able to predict birch pollen concentrations that cause allergic symptoms at all subjects (> 90 pollen m^−3^) with the probability of detection at 0.7, BIAS at 1.2, and success ratio at 0.6. For the year 2016, the probability of detection of high birch pollen concentrations (≥ 75, ≥ 90, ≥ 155) is low and does not exceed 0.35.

A summary of the standard performance statistics is given in Table 5. The correlation coefficients between modelled and observed birch pollen concentrations are similar for both years and equal to 0.34 and 0.31, for 2015 and 2016, respectively. MB shows that the model overestimates the observed birch pollen concentrations for the year 2015 and underestimated them for 2016. FB indicates that the underestimation in 2016 (FB = − 1.43) is higher than the overestimation in 2015 (FB = 0.10). The fractional absolute error is also higher for 2016 (FAE = 1.65) compared to 2015 (FAE = 1.45).

**Table 5:** Mean statistics based on daily birch pollen observations over Poland. Units for MB and MAE are pollen m^−3^; other statistics are dimensionless

Year	Mean observation	Mean model	MB	MAE	*R*	FB	FAE
2015	73.23	133.71	60.48	117.27	0.34	0.10	1.45
2016	551.72	91.02	− 459.71	500.07	0.31	− 1.43	1.65

**Fig. 7:**
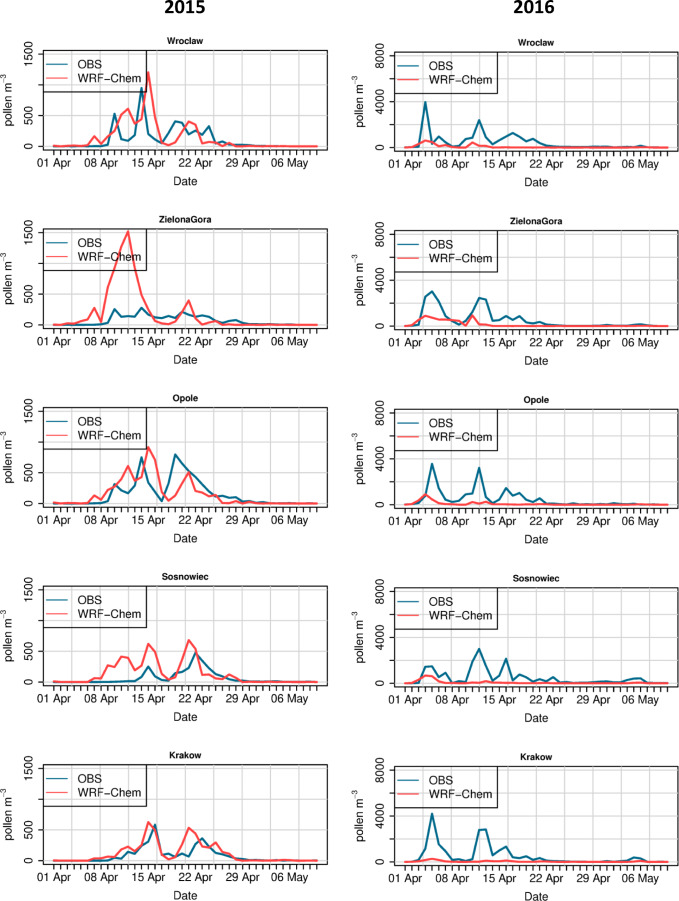
Time series of daily modelled and observed birch pollen concentrations for the year 2015 (left) and 2016 (right). Stations from western and southern Poland

**Fig. 8:**
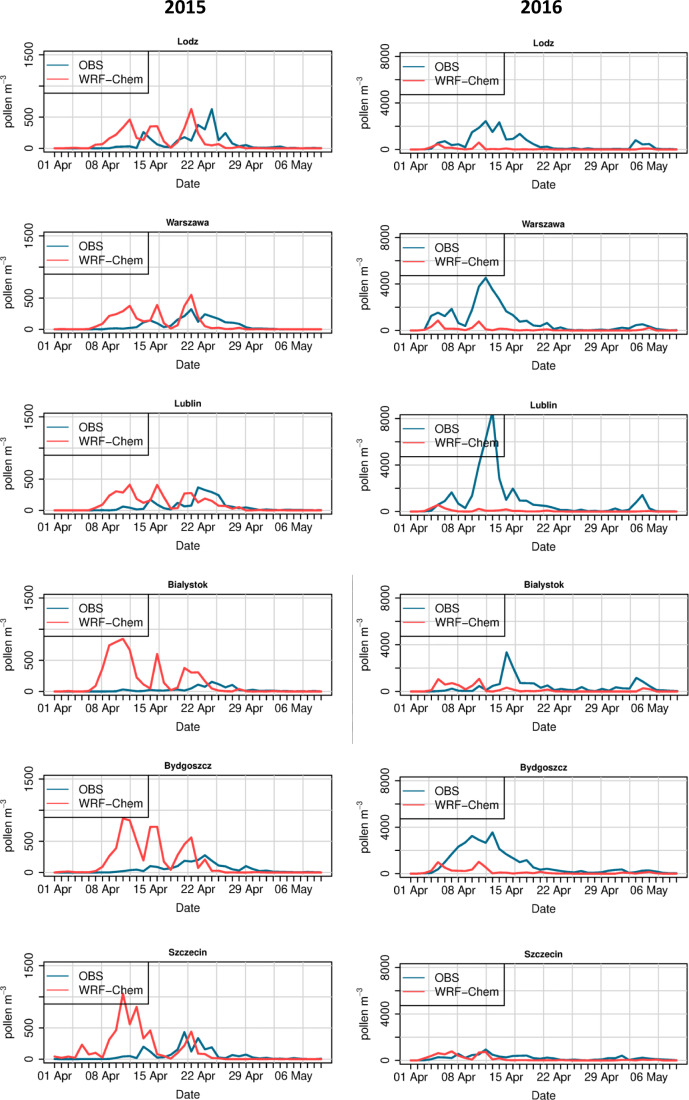
Time series of daily modelled and observed birch pollen concentrations for the year 2015 (left) and 2016 (right). Stations from central, eastern, and northern Poland

Modelled and observed time series of birch pollen concentrations are shown in Figs. 7 and 8. For the year 2015, the WRF-Chem model correctly calculates the time of the first biggest peak of birch pollen concentrations for stations located in southern Poland (Wrocław, Kraków, and Opole). For some stations, i.e. Sosnowiec, Łódź, and Lublin, the model’s first peak is too early compared to the observed due to the too early start of the season calculated by the model. In the year 2015, there are also two stations (Bydgoszcz and Szczecin) for which modelled concentrations are highly over-predicted. For the year 2016, the time of the first peak is in general calculated by the model correctly; however, the concentrations are significantly underestimated for all stations.

## Discussion

The WRF-Chem model with emission parameterization based on heating degree days managed to calculate the main characteristics of the birch pollen season in Poland. In most of the stations, the start of the season was calculated with an error not larger than 3 days in the year 2015 and smaller than 2 days in the year 2016. The spatial distribution of the season’s start in 2015 is in agreement with the general pattern of the vegetation season in Poland, with the latest start in the mountainous area. This pattern is not replicated in the year 2016, which can confirm that the beginning of the season in Poland was under the influence of long-distance atmospheric transport (Puc et al. 2016). In case of the season’s end, the model was closer to the observations for the year 2016—in most of the stations, the difference was not bigger than 2 days. For the year 2015, the mean difference between the modelled and observed season’s start was 9 days.

The study by Sofiev et al. (2015) shows that the ensemble approach (seven regional models) has captured the season onset over Europe for the year 2013 with an error of a couple of days. The mean error for the season’s start from all stations for the individual model did not exceed 5 days. In case of the end of the season, they have shown that the error usually stays within 5 days but can also reach several weeks, especially in the mountainous regions (e.g. Alps). In our study, the biggest difference between the modelled and observed end of the season was minus 17 and minus 18 days for 2015 and 2016 respectively, which means that the end of the season calculated by the model was earlier than shown by observations. For the other stations, differences for both years were also negative or equal to 0. Due to the fact that the end of the season in our model is described via the open-pocket principle, the too-early end of the season could be related to the following: (1) too early start of the season, (2) model overestimating the first peaks in the season, and (3) too low birch productivity assumed in the model. In these situations, the initially available amount of pollen is completed too early in the model, which affects the time of the season’s end. However, the end of the season is a less important parameter, as it has not so relevant impact on the modelled concentrations in the main season such as the date of the season’s start (Sofiev et al. 2015).

Seasonal pollen integral was overestimated by the model for the moderate birch pollen season (2015) and underestimated for the season with high birch pollen concentrations (2016). Underestimation for the year 2016 was much higher than overestimation for 2015. The SPIn modelled values for the year 2016 were 3 times lower at 11 (out of 12 stations) and 10 times lower at 2 stations (Kraków and Lublin). Sofiev et al. (2015) showed that chemical transport models better reflect the SPIn for moderate pollen seasons than seasons with relatively low or high concentrations. They also emphasize that currently, there is no model for year-to-year variation in birch productivity. The total amount of pollen stored in catkins depends on the previous year’s summer and, to some extent, the following winter conditions (Tseng et al. 2020), which are not included in the current emission model developed for chemical transport models. Previous studies have also shown that year-to-year variability in airborne *Betula* pollen concentrations is related to the biennial cycle of pollen production (Spieksma et al. 2003; Grewling et al. 2012; Kubik-Komar et al. 2019). This cycle has been observed at most of the Polish sites (Latałowa et al. 2002; Grewling et al. 2012; Malkiewicz et al. 2016; Kubik-Komar et al. 2019). However, it was also shown that the biennial cyclic rhythm can be interrupted by asynchronous years or the intensity of pollen seasons can be similar during consecutive years (Grewling et al. 2012; Piotrowska and Kubik-Komar 2012), which is a challenge for chemical transport models. On the other hand, the underestimation of observed concentrations by chemical transport models is well known from many studies related to chemical air pollution (Tuccella et al. 2012; Forkel et al. 2014; Werner et al. 2018). This underestimation is linked with the averaging of primary emissions and then concentrations across the model grid, which is compared with monitor locations that might be more influenced by local emission sources than the grid average (Dore et al. 2012). This relation, i.e. the distance between the birch pollen emission sources and the observational site, and additionally the grid size will also have an impact on the model performance for birch pollen concentrations.

Validation of chemical transport models for birch pollen over Europe has been done by Sofiev et al. (2015) and Kurganskiy et al. (2020). Sofiev et al. (2015) presented the results for six individual models and for the ensemble approach based on comparison of the modelled concentrations with stations located in 11 European countries for the year 2013. The correlation coefficient (mean for all stations) for individual models varied from 0.28 to 0.41, while the mean from all models was equal to 0.38. Kurganskiy et al. (2020) ran the Enviro-HIRLAM model over Europe and validated the results against stations located in Finland, Russia, and Denmark for the year 2006. They ran three types of simulations: (1) a standard run, (2) a run that includes a correction for 2-m air temperature based on observational data, and (3) a simulation that includes both the correction for temperature and a grid-based scaling factor for pollen emission based on pollen observations. For each simulation type, they also used three different types of birch cover data. The results for the second and third type of simulations are shown in this paper. The correlation coefficient varied from 0.42 to 0.59 for simulations with T2 bias correction (the value depended on the birch cover map), and from 0.68 to 0.72 for simulations with scaling factor for pollen emissions. FAE varied from 0.81 to 1.21 and from 0.75 to 0.77 for the second and third type of simulations, respectively.

Our study presents the results for different years and configurations of observational stations than the study of Sofiev et al. (2015) and Kurganskiy et al. (2020). Therefore, we should not directly compare the statistics between these modelling results. Our results show that the model’s performance can vary a lot, especially in terms of MB or MAE for 2 years with different pollen seasonal characteristics if no emission correction is used. The correlation coefficient for our results is at a similar level (0.34 and 0.31 for 2015 and 2016, respectively) to that presented in Sofiev et al. (2015). The fractional absolute error (1.45 for 2015 and 1.65 for 2016) is similar to the results presented by Kurganskiy et al. (2020) for their run with T2 bias correction included. Their work, however, has shown a significant improvement in the modelled birch pollen concentrations after application of a grid-bases scaling factor for pollen emission based on pollen observations. Sofiev (2019) has also suggested that a lasting improvement in pollen modelling can be obtained if assimilation targets the emission parameters. His experiment confirmed that correction of the total seasonal pollen emission is possible, efficient, and has a positive impact on the whole modelled season. The experiment, however, used the whole-season data and thus this solution cannot be used, for example, in operational forecasting (Sofiev 2019).

The statistical model for predicting high *Corylus* (hazel), *Alnus* (alder), and *Betula* (birch) concentration levels, based on long-term observations and gridded meteorological data, was developed for Poland by Nowosad (2015). This model has provided satisfactory results for birch and was able to correctly predict 88% of high pollen concentrations, defined as first allergy symptoms during exposure (≥ 20 pollen m^−3^). This type of models requires a relatively dense observational network and long-term time series of observations as input. The deterministic WRF-Chem model used here predicted the first symptoms (≥ 20 g m^−3^) with probability of detection at 0.78 and a success ratio at 0.75 for the moderate birch pollen season in 2015. The results were worse for the season with high birch pollen concentrations (year 2016). This shows that the WRF-Chem model can extend the set of tools used for pollen modelling in Poland. That being said, more effort is needed to improve the model’s performance for the years with high pollen productivity.

The WRF-Chem model was able to reproduce the timing of the first peak of birch pollen concentration in the season at stations in southern Poland for the year 2015 (i.e. Wrocław, Opole, Kraków). For other stations, this first peak is over-estimated, which has further impact on the SPIn value and modelled end of the season. For the year 2016, the model underestimates the observed concentrations during the whole season. This indicates the potential role of correcting the total seasonal pollen emission in improving the model’s performance, as suggested by Kurganskiy et al. (2020) and Sofiev (2019).

Previous studies on PM modelling for Poland have shown that the WRF-Chem model’s performance can be improved significantly even after assimilation of observational data only at the start of simulation (Werner et al. 2019b). This is especially relevant for forecasts for which initial conditions have a great impact on the modelling results for hours that follow. The rapid development of automatic methods in pollen counting (Šaulienė et al. 2019) offers a great opportunity for application of data assimilation methods for pollen modelling in the future.

## Summary and conclusions

In this study, we developed the WRF-Chem model towards birch pollen modelling and then ran the model with birch pollen emission calculated with parameterization proposed by Sofiev et al. (2013). This combination of models has not been tested before. The simulations were run for two seasons: (1) season 2015, which was moderate in terms of birch pollen concentrations, and (2) season 2016, which was characterized by high birch pollen concentrations. The simulations were run over Central Europe and validated against 11 observational stations located in Poland. The results show that there is a big difference in the model’s performance between the moderate (year 2015) and high birch pollen season (year 2016). In general, the model overestimates SPIn and birch pollen concentrations for the moderate season and highly underestimates birch pollen concentrations for the year 2016. For the year 2015, the model was able to predict birch pollen concentrations for the first allergy symptoms (above 20 pollen m^−3^) as well as for severe symptoms (above 90 pollen m^−3^) with a probability of detection equalling 0.78 and 0.68 and a success ratio equalling 0.75 and 0.57, respectively. The model failed to predict these parameters for the year 2016. The results indicate the potential role of correcting the total seasonal pollen emission (2019) in improving the model performance for specific years in terms of pollen productivity. Prediction of high birch pollen concentrations is very important, as birch pollen have a significant impact on the quality of life and productivity of allergy sufferers. Prediction models, for example, can allow allergy sufferers to undertake the appropriate treatment (Nowosad 2015; Nowosad et al. 2016).

It has been emphasized in previous studies that the combined exposure to chemical and biological air pollutants may also strengthen (both synergistically and additively) allergic reactions (Baldacci et al. 2015; Grewling et al. 2019). Therefore, application of chemical transport models such as WRF-Chem for pollen modelling provides a great opportunity for simultaneous simulations of chemical air pollution and allergic pollen with one goal, which is a step forward for studying and understanding the co-exposure of these particles in the air.
